# The multidisciplinary, theory-based co-design of a new digital health intervention supporting the care of oesophageal cancer patients

**DOI:** 10.1177/20552076211038410

**Published:** 2021-09-28

**Authors:** Kristi Sun, Henry Goodfellow, Emmanouela Konstantara, Alison Hill, Debby Lennard, Elizabeth Lloyd-Dehler, Muntzer Mughal, Kathy Pritchard-Jones, Chris Robson, Elizabeth Murray

**Affiliations:** 1 Research Department of Primary Care and Population Health, 4919 University College London, UK; 2 University College London Hospital, UK; 3 North Central and East London Cancer Alliance, 8964 University College London Hospitals NHS Foundation Trust, UK

**Keywords:** Digital health General, cancer, disease, technology, general, gastroenterology, medicine, digital, general, oncology, medicine

## Abstract

**Objective:**

Oesophageal cancer patients have complex care needs. Cancer clinical nurse specialists play a key role in coordinating their care but often have heavy workloads. Digital health interventions can improve patient care but there are few examples for oesophageal cancer. This paper aims to describe the multidisciplinary co-design process of a digital health intervention to improve the experience of care and reduce unmet needs among patients with oesophageal cancer.

**Methods:**

A theory-based, multi-disciplinary, co-design approach was used to inform the developmental process of the digital health intervention. Key user needs were elicited using mixed methodology from systematic reviews, focus groups and interviews and holistic need assessments. Overarching decisions were discussed among a core team of patients, carers, health care professionals including oncologists and cancer clinical nurse specialists, researchers and digital health providers. A series of workshops incorporating a summary of findings of key user needs resulted in the development of a minimum viable product. This was further refined after a pilot study based on feedback from end users.

**Results:**

The final digital health intervention consists of a mobile app feature for patients and carers connected to a dashboard with supporting additional features for clinical nurse specialist. It contains a one-way messaging function for clinical nurse specialists to communicate with patients, functions for patients to record weight and holistic need assessment results which could be viewed by their clinical nurse specialists as well as a library of informative articles.

**Conclusions:**

The multidisciplinary co-design of a digital health intervention providing support for oesophageal cancer patients and health care professionals has been described. Future studies to establish its impact on patient outcomes are planned.

## Introduction

Oesophageal carcinoma is the seventh leading cause of cancer-related mortality in the UK with around 7900 deaths per year.^1,2^ There are around 7500 new cases diagnosed each year, mostly in its late stages,^1^ and the 5-year survival is 15.7% in England.^3^ Management of oesophageal cancer is highly complex with key decision making undertaken using a multidisciplinary team (MDT) approach.^4^ In a drive to improve outcomes, there has been centralisation of surgery at high volume specialist centres.^5,6^ However, the pathway is complex with diagnosis and different components of treatment spread across primary care and local hospitals.^7^ With involvement from numerous health care professionals (HCPs) across different sites, it often falls to the clinical nurse specialists (CNSs) to act as a central point of contact in providing continuity of care for the patient and to coordinate between the various teams.^7,8^

The diagnosis and management of oesophageal cancer have a significant impact on patients’ wellbeing and livelihood. Patients may undergo extensive, life-changing surgery with high postoperative morbidity.^9^ They often require extensive support for nutritional needs after their surgery but also have complex psychological, emotional, spiritual and social needs.^10^ However, surgical resection with curative intent, where surgery is performed to fully remove all cancerous tissue, is only appropriate for 19% of patients at diagnosis.^1^ Most others undergo combinations of radiotherapy, chemotherapy and endoscopy with curative or palliative intent for relieving symptoms.^9–11^ These patients also have complex needs that require support.^9–11^ Guidelines on caring for patients with cancer emphasise the need for a holistic approach that addresses patient-specific needs and improving patients’ quality of life at all stages of the patient pathway.^12^ In fact, at the initial conceptual stages of the integrated cancer system ‘London Cancer’ based in London, a formal network of care providers collaborating to establish cancer patient pathways; a large focus was placed on defining the top ten areas that cancer patients felt mattered most to them.^13^ Highlighted areas included good communication with access to information specific to patients’ needs alongside a holistic individualised approach to patient care.^13^ Despite this, 36%–48% of cancer patients report unmet needs for support.^14^ Holistic needs assessments (HNAs) are recommended to help identify patient support needs and allow HCPs to work with patients to develop patient-specific care plans to address them.^15^ One example is the electronic HNA developed by Macmillan, one of the UK's largest charities devoted to improving care and outcomes for patients with cancer. ^16,17^ It has 48 questions covering five domains: physical, practical, social, emotional and spiritual needs.^16,17^ The recent 5-year strategy plan set out by the independent cancer taskforce to improve cancer outcomes in the National Health Service (NHS) details that every cancer patient by 2020 should have access to an HNA and a care plan at various points in their cancer journey.^18^

In the new digital age with the widespread usage of mobile phones,^19^ patients often turn to the internet and mobile apps to gather information regarding their symptoms, illnesses and for advice in managing their health.^20–22^ Digital health interventions (DHI) have the potential to improve the delivery of health care including streamlining its efficiency and accessibility and providing ways to personalise health care.^23^ Studies have shown that accessing online information prior to appointments can help with information processing and encourage active engagement during consultations, leading to better patient outcomes.^24^ Improvements in technology have led to an influx of health care related apps in the digital market.^25^ Within the cancer field, apps have been designed for a variety of purposes including providing information for patient education, monitoring symptoms and medications, promoting exercise and healthy eating behaviours and offering support for pre- and post-operative surgical care.^26–29^

Despite many mobile health (mHealth) apps, including cancer-related apps, being downloaded each year, many are poorly used, with little evidence of benefit.^30,31^ This may be related to poor development and design processes, with inadequate input from the target audience, resulting in a failure to identify and meet user requirements.^32^ Data suggest that many such apps do not involve HCP expertise in their development,^33^ have little or dubious scientific basis to their content,^34,35^ and lack evidence of their effectiveness and safety.^29^ The poor use of theoretical frameworks may also be a contributing factor,^29,36^ as studies have shown interventions with a theoretical basis are significantly more effective compared to those without.^37^ Apps aimed at patients with breast cancer are the most common cancer-related apps, with other cancers, such as oesophageal cancer, underrepresented.^29,38^ Many existing apps focus on providing generalised information,^35^ with only a few focussing on the quality of life measures including psychological impact or well-being assessments.^39^ In addition, recent reviews have shown that apps tend to be aimed either at HCPs (e.g. guidelines, drug referencing, decision support making tools),^35,40,41^ or patients and the general population (e.g. patient information, self-monitoring tools) separately.^35,41^ There appears to be a lack of products incorporating both HCP assistance and patient assistance tools that could be used by both groups.^35^ This is despite a recent survey showing a majority of HCPs would support the use of an oncological app as a complement to current patient management and would find functions such as assessing the quality of life and visualising patient inputs such as side effects useful.^42^

Difficulties in the implementation of health care technologies within existing current health care systems in the UK have also been highlighted and important implementation factors suggested in a recent systematic review include consideration of how new technologies will fit into current pathways and involving patients in the innovation process.^43^

The design process of DHIs can vary. The Medical Research Council (MRC) has introduced a framework for the development of complex interventions through to evaluation and implementation, with new updated guidance soon to be published in the imminent future.^44,45^ It details how interventions should be evidence based with the use of literature reviews, involve the use of theoretical models, and be evaluated to assess its effectiveness.^44^ User-centred design is a popular methodology that focuses principally on gauging and addressing the needs of the end-user in the development of complex interventions.^30,46,47^ Determining end-user needs can be done through focus groups, interviews and field studies as an example.^30^ Co-design builds on the principles of user-centred design with full involvement of target users alongside designers and researchers across each stage of the design process.^48^ Users play a more active role in comparison to their role in user-centred design where they passively provide input through interviews or as an object of study.^49^ The principles of user-centred design and co-design have been described in the development of many DHIs.^24,29,32,47^

A recent systematic review highlighted the lack of detailed descriptions of the developmental process for cancer apps, however, making it difficult for others to follow on from previous work.^29^ Describing the developmental process is vital in contributing to advancements of the medical field,^50^ and is an example of good practice as highlighted by the MRC framework.^44^ It allows others to replicate processes, evaluate aspects that made it effective and can act as a template for the development of future DHIs for other conditions.^50,51^

We have designed a new DHI that hopes to address an area of high need by specifically targeting oesophageal cancer patients. We hypothesised that a multidisciplinary co-design process would create a functional and valuable DHI and allow us to overcome barriers of poor user engagement and implementation into the NHS.

The aims of this paper are to describe the multidisciplinary co-design process and features of a DHI targeting oesophageal cancer patients.

## Methods

### Design

**Figure 1.**
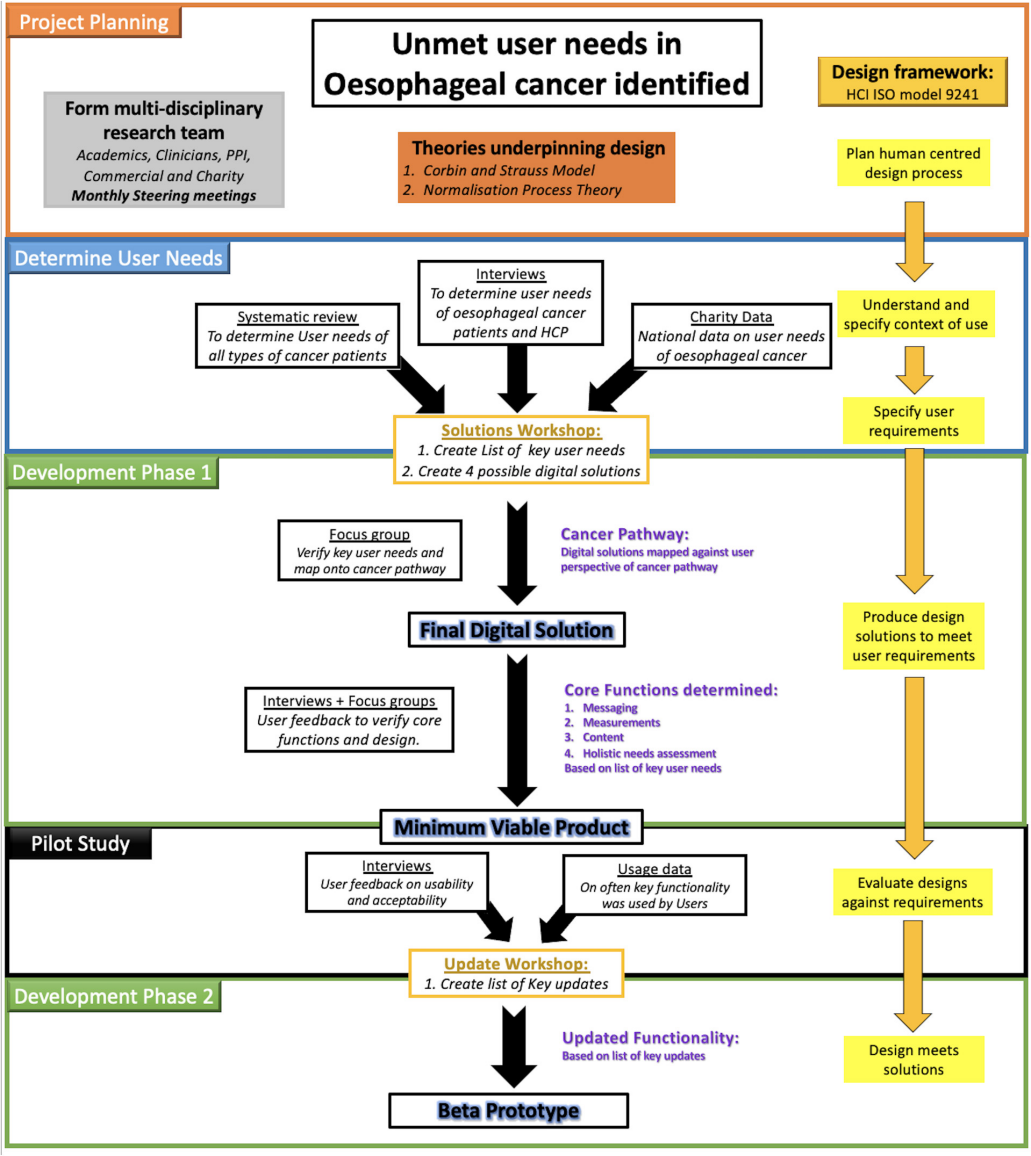
Summary of overall development process. HCP: health care professional, HCI ISO model: human–computer interaction International Organization for Standardization model, PPI: patient and public involvement representatives.

Mixed method, multidisciplinary co-design approach based on the human–computer interaction International Organization for Standardization (ISO) model 9241 was used.^52,53^ A MDT approach was undertaken involving patient and public involvement (PPI) representatives, HCPs, digital software developers from the company Living With,^54^ and researchers with backgrounds in digital health, behavioural science and medicine. The potential benefits of interdisciplinary collaboration have been described previously in offering different skill strengths and broadening perspectives to improve quality, usability and evidence base behind DHIs.^55,56^ Monthly steering meetings were conducted with the full MDT to guide the process and to inform key decisions throughout. The PPI group were recruited through advertising via local hospital and university networks and referrals from HCPs. One representative was known to the research team previously. PPIs were involved extensively throughout the study and contributed to all aspects of the work documented below, including facilitating focus groups, analysing participant feedback, attending meetings and aiding in the recruitment of other PPIs. A co-design approach was used to advocate for a focus on key user needs and enhance end-user engagement with PPI members attending all workshops in the developmental process. We used two theoretical frameworks: Corbin and Strauss and Normalisation process theory to underpin the theoretical design aspects. An overview of the design process is shown in Figure 1.

#### Digital developmental design model

We used previously well-established and standardised techniques from industrial design and software development in our modelling to design a usable end product.^57,58^ ISO model 9241 is a set of international guidelines that focuses on enhancing usability through the use of human-centred design for interactive systems.^52,59^ The components that are incorporated into the system development life cycle include understanding and specifying the context of use, specifying user requirements, producing design solutions to meet user requirements and evaluating design solutions against requirements,^59–61^ as shown in Figure 1 (yellow boxes).^52^

### Theoretical frameworks

#### Corbin and Strauss model of the work of living with a chronic illness

Corbin and Strauss theorised that there were three key components in the management of chronic illness for a patient: ‘Illness work’, ‘Everyday life activities’ and ‘Biographical work’.^62,63^ Illness work refers to managing the condition itself and associated symptoms, reducing complications and handling disability.^62,63^ Managing ‘Everyday life activities’ refers to making adaptations in order to allow a person to continue activities of daily living.^62,63^ ‘Biographical work’ refers to helping patients cope with the feeling of change in their identity from the effects of their illness on their social life, relationships and level of functioning.^62–65^ This model explained the findings of the systematic review we undertook, as described below,^66^ to inform the work presented here, and also resonated with the de novo qualitative research we undertook with patients. Hence though there is some debate as to whether cancer is qualitatively different from other long-term conditions,^67^ we found this the most useful model for conceptualising the overall needs of patients with oesophageal cancer. We used the three lines of work throughout the developmental process to create a framework around which a list of key user needs was generated. This then went on to underpin the core functionalities of the DHI.

#### Normalisation process theory: The work of implementation

Normalisation process theory (NPT) is a sociological theory that focuses on the work required to implement, embed and integrate (or ‘normalise’) new practices or technologies into routine health care.^68,69^ NPT posits that the four factors which influence the normalisation of intervention are: coherence, cognitive participation, collective action and reflexive monitoring.^68,69^ Applying NPT to the development process of the DHI led to the understanding that uptake and use would be promoted by the following points: patients and HCPs understanding the focus of the intervention and its relevance to them (coherence), being committed to personally investing in it (cognitive participation), feeling supported in using the intervention and that it could be easily incorporated into their daily life without the need to overcome many complex barriers (collective action) and to feel from their own assessment that it is worthwhile (reflexive monitoring). Our work focussed on targeting these four factors to overcome the translational gap from research work to the clinical setting (Supplemental Appendix 1).

### Determining end-user needs

#### Overview

The first step of the project involved establishing the key end-user needs, which we did through three complementary approaches, described in more detail in the following sections. First, a systematic review was done to synthesise evidence from the known literature to determine the general needs of all cancer patients.^66^ Second, as our end-users included patients with oesophageal cancer, their carers and HCPs involved in the care of patients with oesophageal cancer, we undertook focus groups with representatives of these target populations to identify the different end-user requirements. Third, we used an anonymised and aggregated data set from Macmillan consisting of a summary of all the reported concerns of oesophageal cancer patients across England over a two year period. The summary of the concerns was collected using Macmillan's electronic holistic need assessment (eHNA).^17^ Finally, a workshop was conducted to synthesise all the collected data and create a list of key user needs. Having three different types of inputs allowed us to triangulate the data and consider which needs were specific to patients with oesophageal cancer, which were more generalisable to the greater cancer population, and which needs should be prioritised.

#### Systematic review

**Figure 2.**
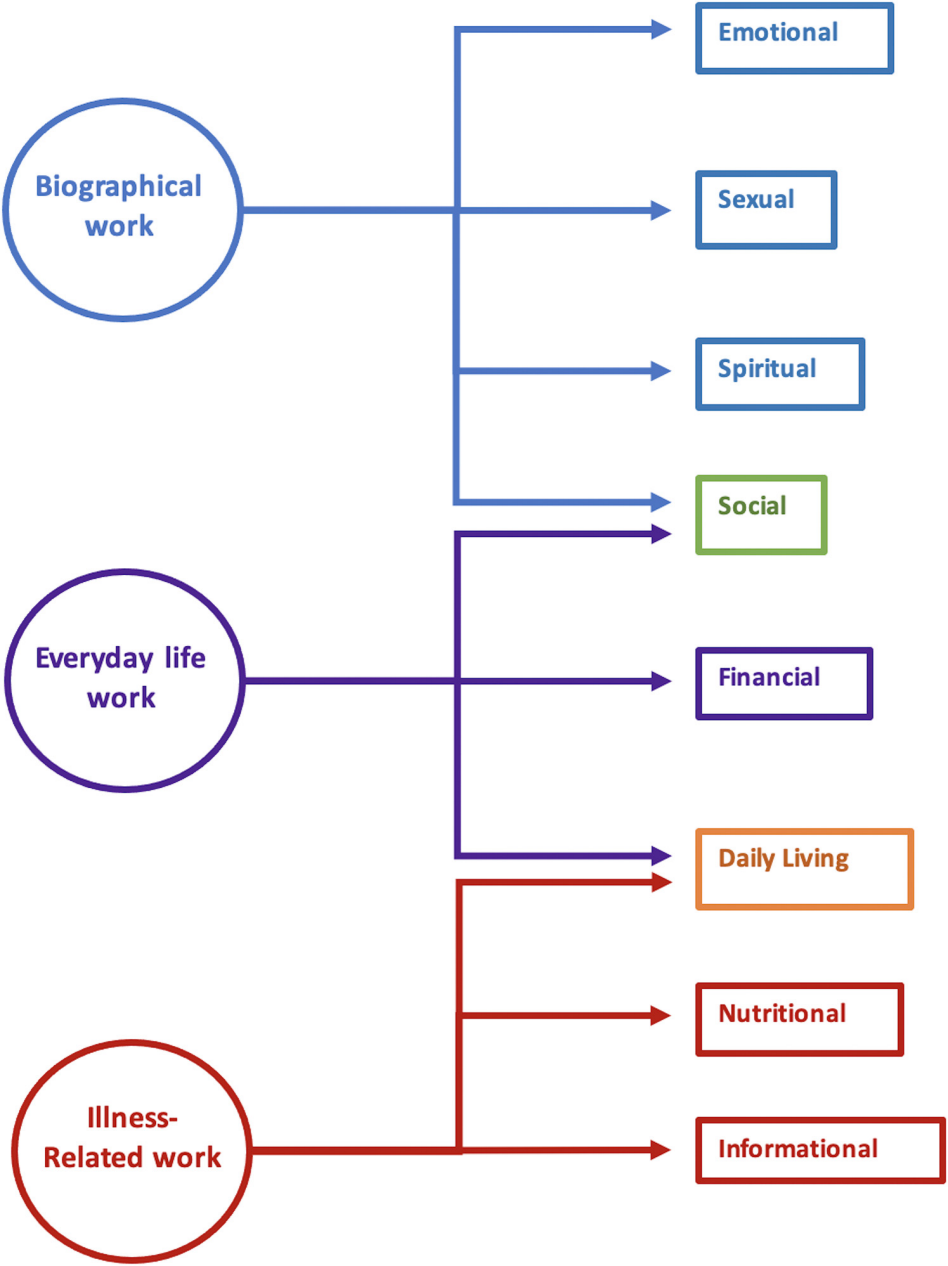
Corbin and Strauss model from Webb et al.^66^

A systematic review was conducted according to Cochrane Methodology to help identify the non-medical support needs of patients with cancer that the DHI would aim to address. Further details regarding methodology and full results have been published in a separate paper.^66^ A total of 46 studies were selected for final analysis after screening to generate a comprehensive list of supportive needs in cancer patients. These needs were grouped into domains as they were first listed in their original article before being categorised together according to the model of Corbin and Strauss (Figure 2).^66^ The use of the model as a framework allowed comparison of perceived importance of categorised needs that were classified under the same type of work. Key findings included confirming patients’ need for ‘high-quality, comprehensible and timely information’ including an understanding of what to expect throughout the treatment pathway, for emotional support and for practical supportive care with regards to everyday tasks. The authors recommend that supportive care interventions should focus on empowering patients to feel involved in their ongoing care.^66^

#### Interviews

To identify specific user needs, interviews were conducted with PPI members with a history of oesophageal cancer and their carers. The seven participants were recruited through PPI networks within universities, cancer charities, hospitals, online advertisements and word of mouth. Inclusion criteria for patients and carers were: >18 years old, reported diagnosis of oesophageal cancer or having cared for someone with a diagnosis, English-speaking and having the capacity to provide consent for being involved in research. Of the seven PPI members interviewed, all were white and middle aged (age range: 50–70 years) and consisted of six people with a previous diagnosis of oesophageal cancer and one carer.

In addition, separate interviews were conducted with seven HCPs from four different hospital sites, to identify their user needs. Inclusion criteria for HCPs were those working at a participating hospital site caring for oesophageal cancer patients. The seven HCPs consisted of one upper gastrointestinal surgical consultant, one dietitian and five CNSs.

Interviews lasted between 30 and 60 min, and were semi-structured, following a topic guide developed by the research team and piloted with our PPI. Questions focussed on understanding the patients’ experience of their cancer, including areas of met and unmet need. Participants were asked what features they would like in a DHI and why, as well as suggesting content of information articles and appropriate presentation which could encourage greater use of the intervention (Supplemental Appendices 2 and 3). Interviews were recorded and transcribed verbatim by a professional transcription company. Data were analysed thematically by two of the co-authors (HG, EK) and themes found in the data were discussed with the entire MDT team.

#### HNA data

Macmillan shared an anonymised and aggregated national data set generated from patients with oesophageal cancer who completed their eHNA,^17^ in 2017 and 2018 (*n* = 2264). The data provided information about the concerns mentioned by patients who had completed the HNA, with concerns ranked in order of how often they were mentioned. The list of concerns was then compared to the concerns found in the literature review and those noted in the focus groups and interviews.

#### Workshop part 1: Synthesising data to on user needs

In order to synthesise all the data collected from previous stages of the study, a workshop consisting of the core research team was undertaken. Findings were integrated with the theoretical models of Corbin and Strauss and NPT to identify a list of key user needs that the intervention needed to address (Table 1).

**Table 1.** User needs categorised according to themes of Corbin and Strauss*.

Corbin and Strauss theme	User need
Illness related work	Information needs • To be able to access a single source of reliable and truthful information • To access further information on their illness and treatment • To know or understand a new uncertainty • To know who to ask that could help answer questions on different topics • To find the right answer for them • To choose when to receive information • To know what is going on* • To understand what and why* Nutritional • To optimise their chances • To develop ‘Survival strategies’ to cope • To know what to do about food and weight • To know what they could do to help*
Everyday life work	Daily living • To know how to help as a carer • To be educated about the importance of exercise Social • To know that their loved ones are cared for Financial • To know how to manage practical issues, for example, with regards to travel, housing and finances. • To be reassured that they have the money to survive
Biographical work	Emotional • To be seen as a whole human being • To have a source of hope • To have someone to talk to • To feel they can ask for help without feeling guilty • To develop ‘Survival strategies’ to cope • To know how to talk about the illness and difficult subjects as a carer • To help them see there is a new beginning • To understand their emotions • To help signpost before being given bad news • To feel normal* • To want to be in control* • To feel cared for in and out of hospital* • To know that they can trust the team* • To feel they are being cared for/to feel safe* Spiritual • To know there is life after cancer

NB*: key user needs identified by core research team.

#### Workshop Part 2 digital solutions:

Once the key user needs were identified from Part 1 of our workshop, four potential digital solutions were devised based on these needs as shown in Table 2.

**Table 2.** Digital solutions paired with user needs it aims to address.

Digital solution	User needs it meets
‘Pocket (CNS)’ specialist • The model is developed in a way for patients to feel that their cancer clinical nurse specialist (CNS) is always with them and can respond to their needs	• To be cared for at every step • To help communication with the medical team • To help communication between HCPs • To provide patients with a source of truth • To know who to ask and when
The ‘If/What’ machine – Question and Answer dashboard • Patients can ask the ‘machine’ their question to find some support and answers • Answers will then be complemented by responses from real CNS	• To help patients understand the new normal, without anxiety • To help patients feel more in control • To help patients to know who to talk to • To help patients see the reality of their situation • To help improve patient trust in their team • To facilitate patients to be able to ask for help without feeling guilty
Patient empowerment – ‘What I can do to help’ • This is a digital solution in the form of an App that would provide patient with the tools and information to help them improve outcomes	• For the patient to know what they can do to help themselves
‘Digital Cockpit’ • This would compare the cancer journey to flying a plane • The cockpit has a guidance system to provide patients with information, e.g. what to eat and drink ‘whilst onboard’, what exercises to do, what could form part of their daily routine etc. • Patients would know where they are on their cancer journey and would be able to know their healthcare teams	• To help patients feel more in control of their livelihood • To help improve patients’ trust in their healthcare team • For the patient to know what they can do to help themselves

### Development Phase 1: Minimum viable product

#### Overview

Phase 1 of the development stage involved developing a minimum viable product (MVP). We started with creating potential digital solutions based on the identified key user needs. A focus group was conducted with oesophageal cancer patients to map these needs against the cancer pathway. User personas for HCPs were created from HCP interviews and patient personas were created from unpublished data from Macmillan. Personas are a common tool used in user-centred design to visually represent the needs of a group of end users through a fictional character.^70^ User personas and findings from the cancer pathway focus group were used to determine the final digital solution that best-addressed user requirements from which to build our MVP. Core functions and DHI content were subsequently developed to create the final product.

#### Cancer pathway focus group

As part of optimising the management of patients with cancer, the local Cancer Alliance had developed patient pathways.^71^ These pathways aimed to combine the strengths of treatment at high volume centres (known to improve outcomes and survival)^71^ with the advantages of care provided as close to the patient's home as possible, i.e. in community, primary or local secondary care settings. The details of the pathway for patients with oesophageal cancer were mapped, including decision points and options for consideration. This map was presented to a focus group of the same PPIs involved previously with the interviews, who considered key information and support needs for each point on the pathway. It was decided that the product would be designed to be introduced immediately post-diagnosis where patients had the highest unmet needs.

#### User personas

Several user personas were created for both HCPs and patients. Personas included information about cancer patients and HCPs’ backgrounds, their use of technology, their goals, behaviours and attitudes and their frustrations with the current system. HCPs personas were created from interviews with CNSs. Patient personas were created by Macmillan using its own unpublished data. The unpublished data were based on cancer patient segmentation data generated using a large survey to group cancer patients into different groups. The different digital solutions were compared to the HCP and patient personas to determine which would be most inclusive. The different digital solutions were compared to the HCP and patient personas to determine which would be most inclusive.

#### Core functions of DHI

Four key functions were developed and tailored according to key user needs that had been verified as seen in Table 3. They were added to the final digital solution during the developmental phase. A messaging system was deemed important in order to allow communication between HCPs and patients and the inclusion of an HNA would allow identification of patient concerns. A function for patient-reported outcome measures (PROMs) such as inputting regular weights was also included from workshop feedback so patients could feel supported at home and allow better monitoring of patients’ health for HCPs. A content library was created to meet key informational needs of patients and support feelings of empowerment. Software engineers from Living With, a digital health company, worked to integrate these functions into a professional-looking and user-friendly digital solution.

**Table 3.** Final core functions of DHI and the user needs it targets.

Final DHI function	User need it addresses
Messaging system for communication between HCPs and patients	To feel cared for in and out of hospital
	To know they can trust the team
	To feel they are being cared for and to feel safe
PROMs: HNA and weight	To want to be in control
	To feel cared for in and out of hospital
	To feel they are being cared for and to feel safe
Content library	To have a source of hope
	To know what their chances are
	To know or to understand this new uncertainty
	To know what is going on
	To understand what and why
	To feel normal
Dashboard (CNS)	To feel cared for in and out of hospital
	To know they can trust the team
	To feel they are being cared for and to feel safe

CNS, cancer nurse specialist; DHI: digital health intervention; HCP, health care professional; HNA, holistic needs assessment; PROM, patient-reported outcome measure.

#### DHI content

The research team developed a set of seven evidence-based criteria to ensure that the contents of the DHI were of high quality (Table 4). All content was obtained from evidence-based sources reflecting current best practice as stated by National Institute for Health and Care Excellence (NICE) guidelines on their *Evidence Standard Framework for Digital Health Technologies*.^72^ Validity of the DHI was ensured through rigorous reviewing process and addressing all key areas in the Mobile App Rating Scale (MARS). MARS is a simple standardised tool designed to evaluate the quality of health apps against four objective measures of engagement, functionality, aesthetics and information quality.^73^

**Table 4.** Criteria for DHI developed by research team.

1. Information Quality	• NICE evidence standard framework for digital health technologies • NHS best practice
2. Language	• English • Level key stage 3 (average UK reading age 13–14 years) verified using the Flesch-Kincaid Grade tool.^74^
3. Tone	• Positive, cheerful, matter of fact, focussing on what can be done
4. Coverage	• Covers the areas identified in the systematic review • Covers the areas identified by users (qualitative work, HNA)
5. Interoperability	• Functions in all operating systems for smartphones and tablets
6. Accessibility	• Use of audio, images, videos
7. Validity	• Rigorous reviewing process • MARS

DHI: digital health intervention; MARS: Mobile App Rating Scale; NICE: National Institute for Health and Care Excellence; NHS: National Health Service; HNA: holistic need assessment.

**Figure 3.**
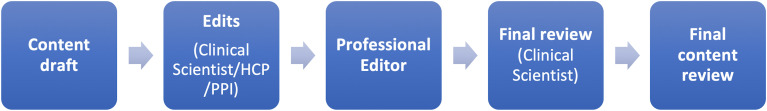
Multi-stage process review of article contents. HCP: health care professional, PPI: patient and public involvement representative.

The initial writing of article contents to support patient needs and concerns was undertaken by various team members according to their respective areas of expertise, e.g. social content by PPI members, psychological needs by those with a background in psychology. The content then underwent a multi-stage review by the rest of the team as shown in Figure 3.

Regular steering meetings were led by the research team to review progress. In order to ensure the created content matched patient needs, focus groups were held with patients and HCPs to review content and pathways regularly.

#### Revisions and creation of MVP

Focus groups and a workshop involving researchers, PPI and HCPs were held to suggest revisions. Feedback was elicited on the presentation and design of the DHI, its name and whether the functions met all of their specific needs. Content was also reviewed to ensure it was readable and easy to understand and suggestions were taken on whether any additions were required. The software engineers and research team made adjustments and incorporated recommendations such as removing medical jargon, which finally led to the creation of an MVP (alpha-prototype).

### Pilot study

A pilot study was conducted over a 6-week period, involving nine participants (six oesophageal cancer patients and their carers, two CNSs and one dietician) to determine the feasibility and acceptability of the MVP. Oesophageal cancer patients, their carers, CNSs and the dietician were recruited through pre-established clinical networks and written consent obtained. Inclusion criteria were patients with oesophageal cancer within London and their carers or those with a history of oesophageal cancer, age > 18 years old with the ability to communicate in English and with access to a smartphone or tablet. Inclusion criteria for HCPs were those looking after patients with oesophageal cancer. Training was provided by the research team who were also readily contactable at any point during the pilot.

Outcome data were collected via two approaches. The DHI itself collected user data that could then be made available to researchers for analysis. User feedback was also collected for qualitative thematic analysis through user interviews, post study focus groups and from comments submitted as anonymous feedback via the DHI. Qualitative data from user feedback highlighted overall good acceptability of the DHI from patients, carers and CNS with most choosing to continue using the DHI, with the exception of one patient who moved their care to a different hospital and hence dropped out of the study. Some issues with usability were highlighted regarding entering patients’ weights and completing HNAs however. Quantitative user data supplied by the app and Living With showed that 97 written articles were viewed a total of 413 times. Findings from the pilot study were used in a subsequent workshop to determine further improvements to the DHI.

### Development phase 2: Beta-prototype

#### Pilot update workshop

A second workshop was organised upon the conclusion of the study which consisted of three researchers, one PPI and two members of Living With. Preliminary results from the study were reviewed and key updates to the app regarding content and functionality were determined to build the beta prototype for the second developmental stage as shown in Table 5. The beta-prototype will be used in future studies.

**Table 5.** User feedback from pilot study and suggested DHI updates.

User feedback	Suggested DHI updates
Linking HNA with articles	• For relevant concern articles to be linked after completion of HNA to allow easy access for patients
Erroneous data – (patients inputting wrong weights or HNAs by mistake)	• To edit the function for weight • To add in functions to record weight in pounds/stone/kilograms • To be able to create draft versions of HNA to save and come back to • To edit functions for the HNA to manage incorrect HNAs
Updates to content article	• To edit original articles based on feedback • For 50 + additional articles to be added
Dashboard alerts	• Dashboard to indicate which patients have added PROMs • Dashboard to alert if PROM has changed significantly
Additional functions	• To explore possibility of adding calendar functions, an organiser and for files to be uploaded and shared/stored

DHI: digital health intervention; HNA: holistic needs assessment, PROM: patient-reported outcome measure.

## Results

### Overview

**Figure 4.**
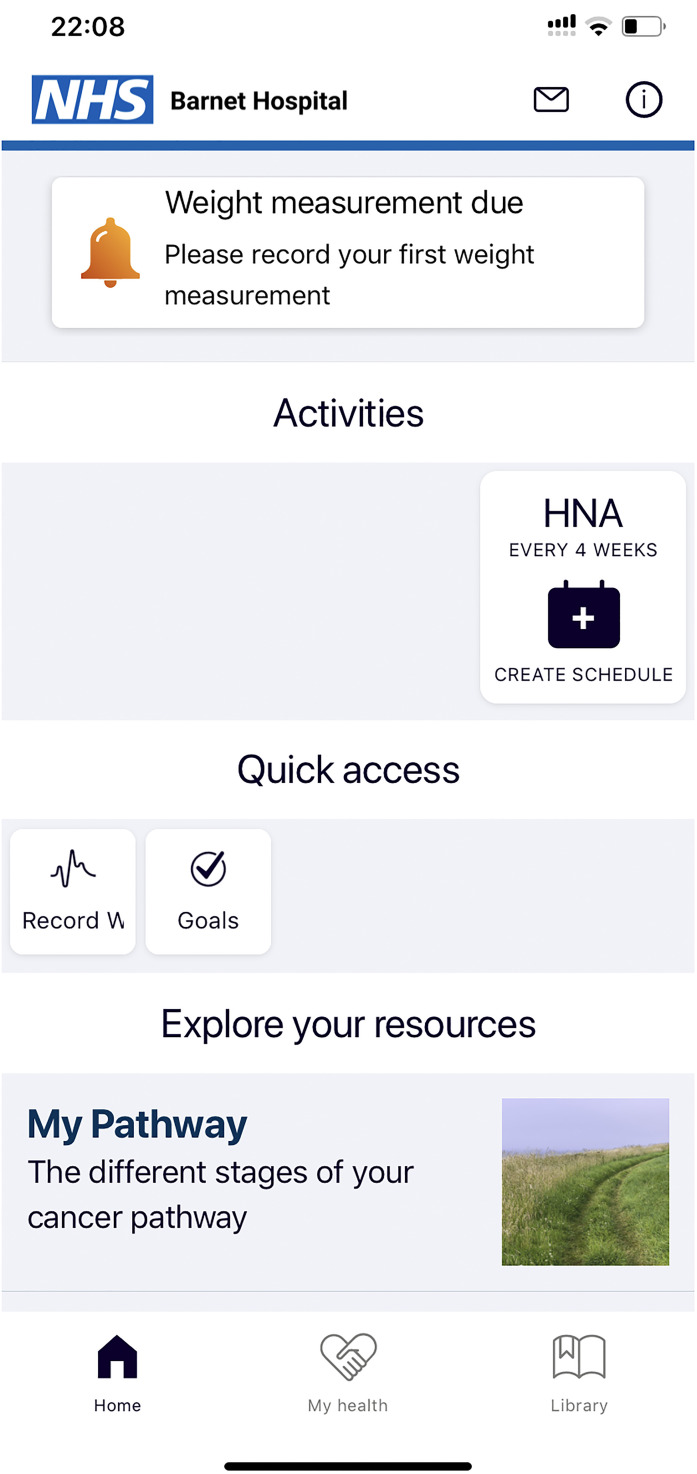
Home screen on the mobile app function of the digital health interventions (DHIs) available to patients.

**Figure 5.**
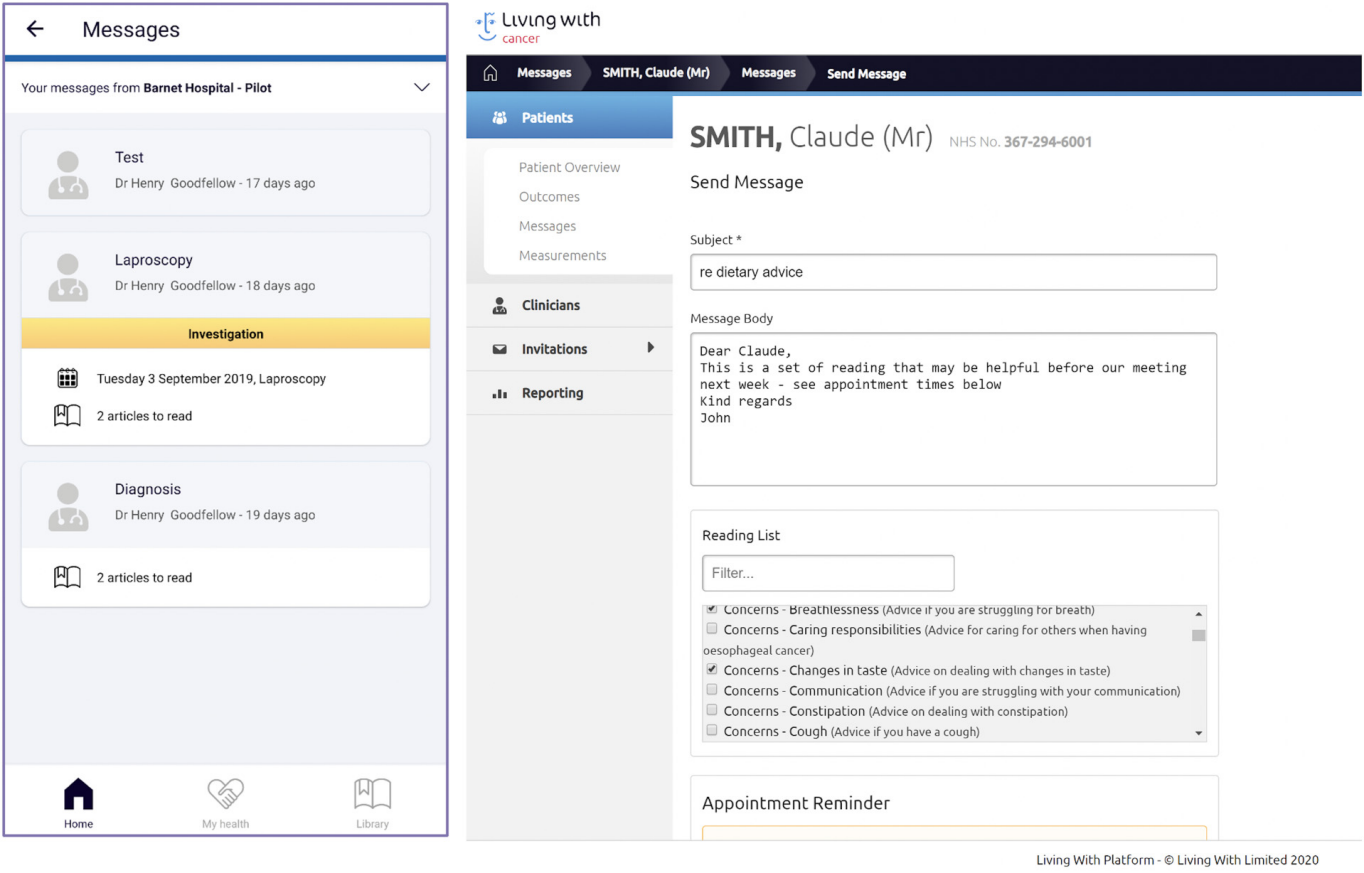
Messaging function.

**Figure 6.**
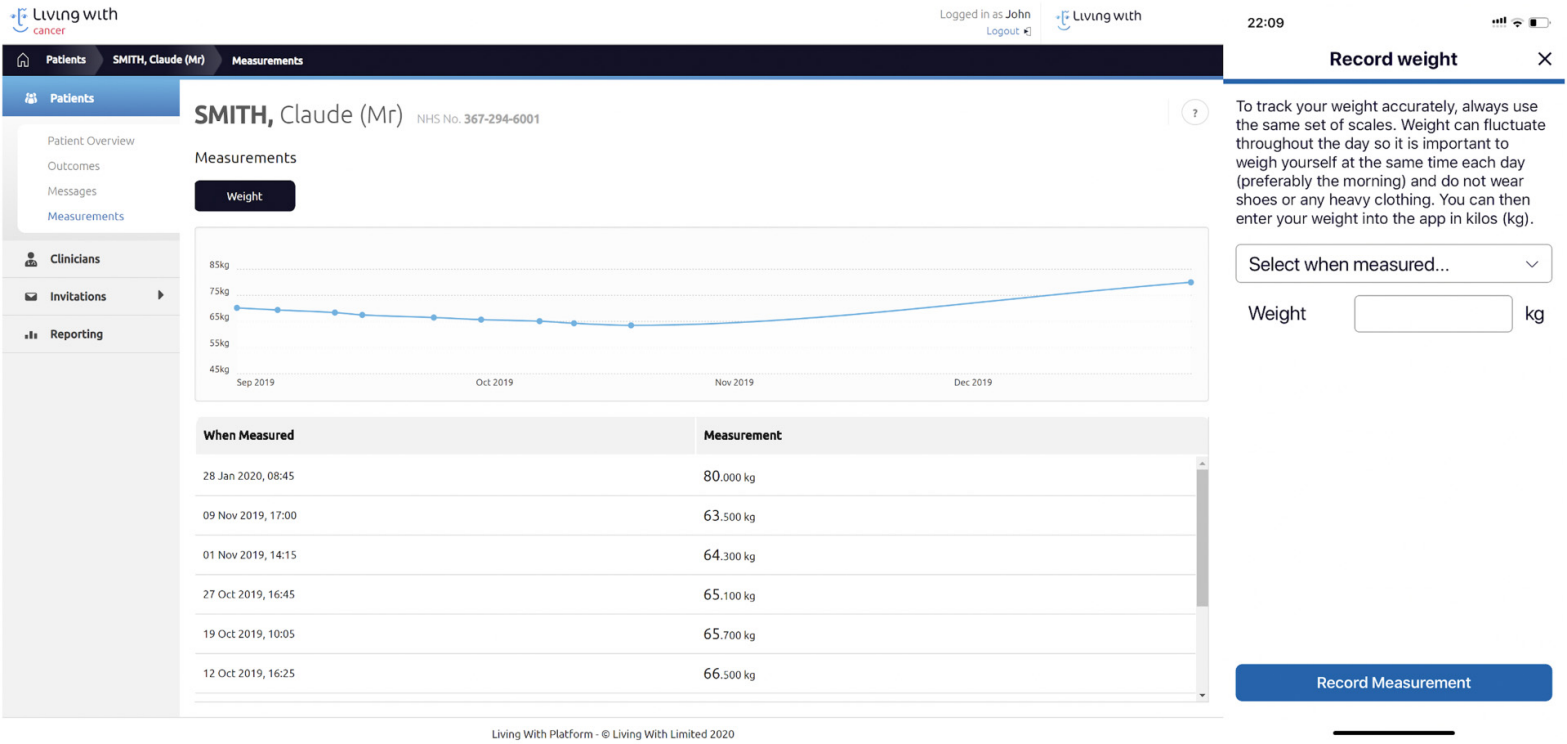
Function for weight tracking.

**Figure 7.**
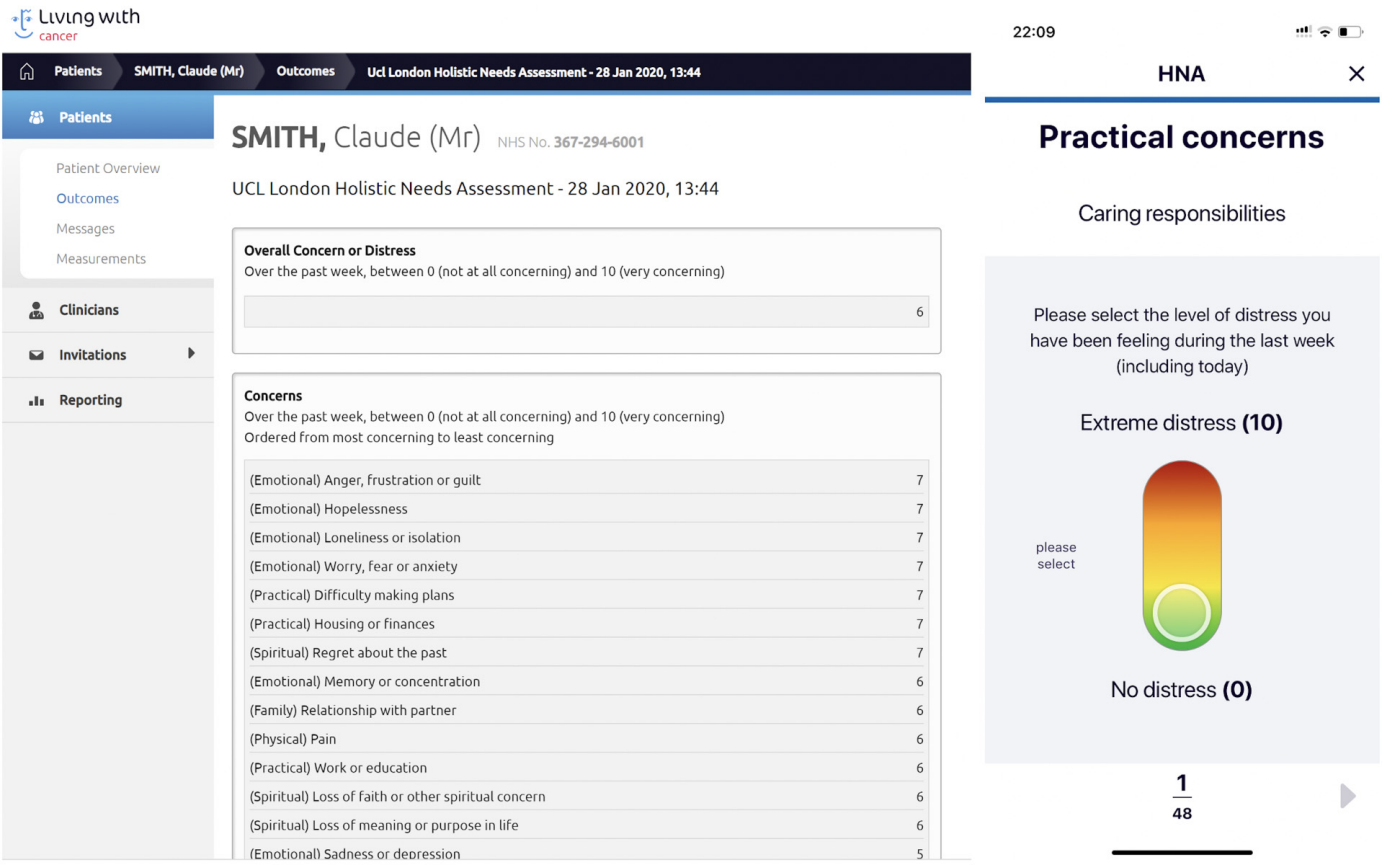
Function for patients to complete HNAs and for CNS to view completed HNAs. HNA: holistic needs assessment; CNS: cancer nurse specialist.

**Figure 8.**
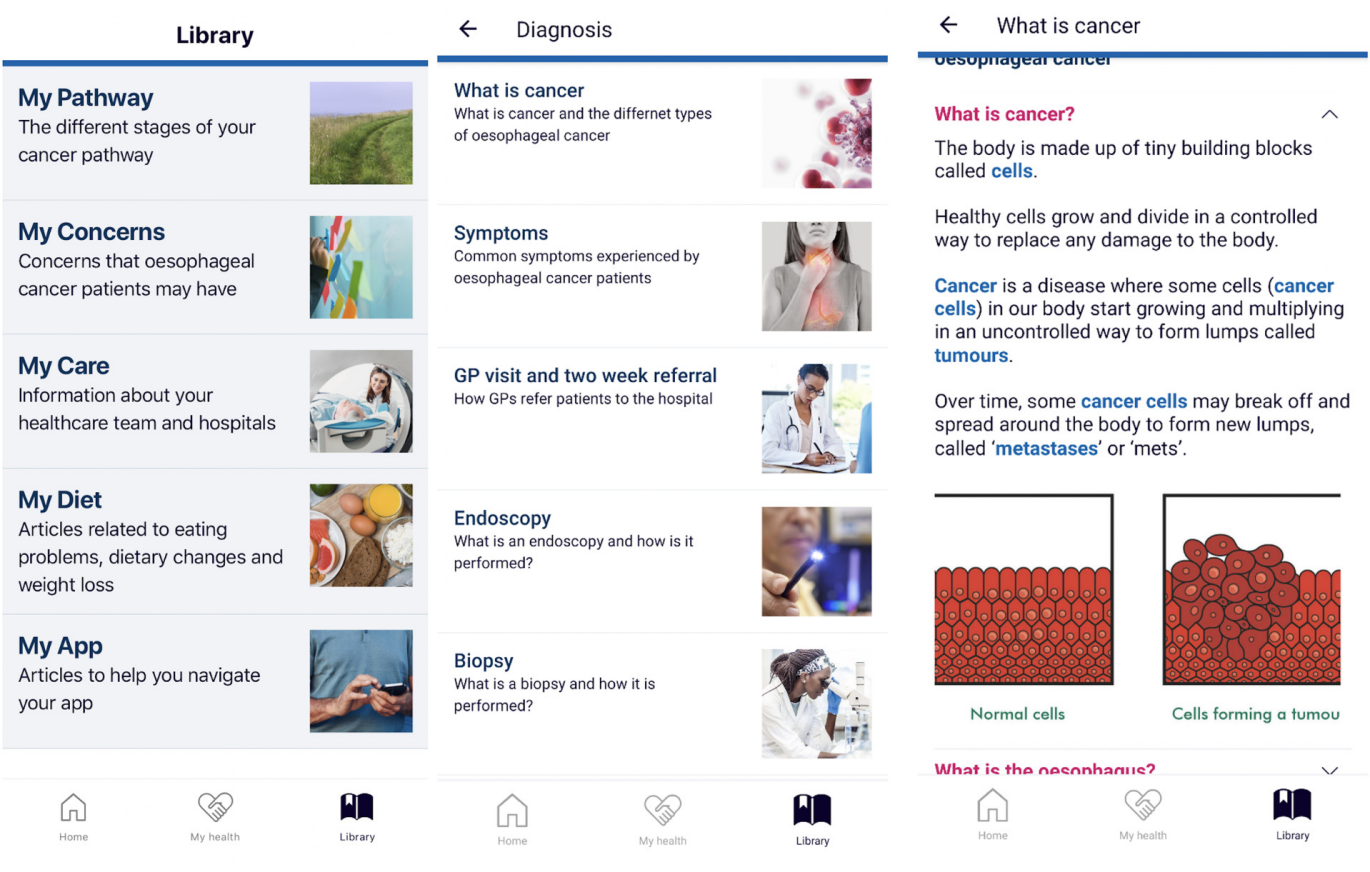
Library content of articles.

The final interactive product is shown in Figures 4 to 8 that interlinks a patient interface with an HCP interface. The patient interface was designed to be an easy-to-use downloadable smartphone application composed of the following main features:

- Home screen.
- Messaging.
- PROMs and goals.
- Library Content (Articles).

Carers at the time were only able to access the app with the patient although future developments may allow for separate carer logins. Tailored to work alongside the app, a dashboard was created as an interface for HCPs to interact with (Figure 5). It consisted of additional features such as sending messages to patients and viewing measurements they had inputted.

### Home screen

The home screen (Figure 4) provided easy navigation to the other four main features. A notification section at the top of the page offered an option to send important reminders to patients, e.g. regarding appointment times and to record measurements if they are due. On the dashboard, CNSs also have the option of viewing a list of patients under their care on the home page.

### Messaging

The ‘Messaging’ section (Figure 5) allowed patients and their carers to view messages sent from CNSs and other HCPs in a one-way messaging system. Examples of messages that could be sent include appointment reminders, links to articles that CNSs feel may be helpful to a particular patient, as well as simple text messages. On the dashboard, CNSs were able to view previous messages alongside messages sent by other HCPs.

### PROMS and goals

The interactive part of the app allowed users to set target goals for themselves and enter data for PROMs. This included regular weekly weights so that patients, carers and CNSs could track their progress (Figure 6). Patients were also able to complete an HNA to identify their main concerns (Figure 7). They were encouraged to submit the HNA at key points on the cancer pathway including at diagnosis, beginning of treatment, post-treatment and discharge. The HNA was based on the London Cancer Holistic Needs Assessment which covers Practical Concerns (e.g. bathing, housing or infancies, grocery shopping), family concerns, emotional concerns, spiritual concerns and physical concerns.^75^ HCPs were able to view submitted HNAs on the dashboard with the hope of allowing these needs to be addressed and considered in the overall management of the patient's care by the healthcare team. HCPs could also track any changes in patients’ weight over time on the dashboard.

### Library

The library included over 100 articles containing supporting information with regards to oesophageal cancer (Figure 8). These were split into five main categories: My Pathway (related to cancer-related investigations, treatment and associated procedures); My Concerns (aimed to address main cancer-specific concerns reported by patients such as symptom management and emotional concerns); My Care (related to patients’ care team and their respective hospitals); My Diet (related to dietary changes, issues with eating and weight) and My App (related to helping patients navigate and use the app). A full list of articles is included in Supplemental Appendix 4.

## Discussion

This paper has described in detail the developmental process of a DHI using theory, multidisciplinary and a co-design approach. Our intervention hopes to address a large area of unmet needs. To the best of our knowledge, there are no current DHIs specifically addressing the needs of oesophageal cancer patients. Our DHI, therefore, has the potential to be of high impact in providing support for oesophageal cancer patients. Given the complexities of the current cancer pathway,^7,8^ it is hoped that this DHI can also reduce CNS workload and improve both patient experience and outcomes. A recent study showed that huge caseloads often limited the care CNSs could provide, in particular with regard to addressing information needs and conducting HNAs.^76^ These are areas covered by our DHI and we have highlighted other important functions of the DHI in this paper and how they were designed from the integration of data from multiple sources.

The multiple different components in our approach were beneficial. Taking an MDT approach, with support from important stakeholders such as cancer charities and HCPs during the development process allowed different perspectives to be taken into account and could also help overcome barriers in implementing it in the future.^43,77^ Endorsement by HCPs provide credibility and reassurance to patients regarding the quality of the DHI which can then encourage greater patient engagement.^78^

We also followed recommendations by the MRC framework in undertaking a theoretical understanding when developing a complex intervention.^44^ The use of NPT allowed us to address and bridge the translational gap from research work to implementing it as part of routine use for patients and HCPs.^68,69^ The model of Corbin and Strauss helped us consider all the relevant factors that impact a patient with a complex medical condition.^62,63^

Our co-design approach was also important. The involvement of end users in co-designing the intervention at each step of the process allowed us to target their precise needs and ensure its relevance and usability which was reflected in the qualitative feedback from the pilot study. We were able to address the specific requirements of both groups of end-users regarding patients and HCPs. For example, the library content was tailored towards informative education for patients. This included providing nutritional support and encouraging physical activity which have previously demonstrated benefits in improving quality of life for oesophageal cancer patients.^79–81^ In addition, the interactive dashboard for CNSs for example, allowed them to track important PROMs, e.g. HNA, weight of patients.

By having both a mobile app feature and a dashboard feature as part of our DHI addressing needs of both types of end users, it is hoped that these features work synergistically to improve the patient experience.

We have also incorporated formal evaluation in our methods as it is another important factor highlighted that can help implementation.^43^ Initial findings from our pilot study provided research-based evidence in the form of both qualitative and quantitative data for the feasibility, usability and benefits of our DHI.

Our study adds further evidence to the existing literature on the benefits of co-design which have been successfully used in the design of other DHIs targeting cancer patients.^49^ However as mentioned before, these studies often do not describe the developmental process at all or with enough information to allow it to be replicated by future researchers.^29^ By describing the developmental process in detail, we hope it can be used as a model example for future research groups when developing new DHIs for cancers and other chronic complex conditions to continue building on and improving current existing practices.

### Limitations

There are a number of limitations to our work. It could be argued that patients and HCPs recruited to our study and focus groups tend to be more engaged with their health and have a greater interest in the use of healthcare related apps. For the development phase, PPIs who were involved were a select group of patients who had received curative treatment for their cancer and had an interest in research. We found it difficult to recruit patients with active oesophageal cancer who were receiving palliative, rather than potentially curative, care. The views of PPIs may therefore not be reflective of the needs and requirements of the greater cancer patient population. However, we do not think this affected the quality of our end product as our pilot study included newly diagnosed oesophageal cancer patients, some of whom had incurable disease. They responded positively to the DHI. There are often concerns regarding the usability of the app with older cancer patients who often have cognitive decline with a decreased level of functioning and hence may have additional difficulties in navigating DHIs.^82^ However, our use of ISO modelling focussing on usability may help offset some of these factors. In addition, 50% patients included in our pilot study were over the age of 70 and they reported good usability of the DHI. We did not use any quantitative measures of usability such as System Usability Scale that could provide a more objective measure for comparison^77^ but this can be considered in future studies.

One group of end users who were not involved in the development process was the Chief Information Officers or members of hospitals’ digital team who could have helped with the future implementation process. However, we have used theories such as NPT to help consider important factors in implementation to try and offset this.

The developmental process is long, costly and labour intensive. We believe this is offset by the careful measures and integrated methods we have taken for a greater likelihood of success and long-term viability in addition to its potential to benefit cancer patients for whom there are large unmet needs. Another limitation is that there is no current evidence this DHI will benefit patient outcomes or that the methodology used will result in higher user engagement when implemented. However, further studies to evaluate this are currently in development.

### Future implications

Further improvements to the beta-prototype are currently being made. Feasibility trials and future randomised controlled trials to determine their impact on patient outcomes are in development. Our ultimate end goal is for its full integration into current NHS IT systems and to expand coverage to other cancers and hence broaden its impact. Although we are still at a very early stage in the developmental process, we hope we have built a strong foundation based on the protocol described in this paper that will greatly increase its chance of success for implementation. It is hoped this DHI can improve cancer patient experiences and their quality of life, reduce CNS administrative workload and improve health service efficiency as well as improving health outcomes.

## Conclusion

This paper provides a detailed description of the multiple sector co-design of a DHI to provide support for oesophageal cancer patients. It aims to address an area of unmet need for oesophageal cancer patients and has the potential to have a high impact on improving patient care and optimising cancer services. It can hopefully be used as a basis from which future studies formally assessing its effects on quality of life and patient outcomes can be developed.
